# Transcriptome profiling of blood from common bottlenose dolphins (*Tursiops truncatus*) in the northern Gulf of Mexico to enhance health assessment capabilities

**DOI:** 10.1371/journal.pone.0272345

**Published:** 2022-08-24

**Authors:** Jeanine S. Morey, Brian C. Balmer, Eric S. Zolman, Ryan Takeshita, Sylvain De Guise, Teresa K. Rowles, Cynthia R. Smith, Randall S. Wells, Lori H. Schwacke

**Affiliations:** 1 Conservation Medicine, National Marine Mammal Foundation, San Diego, California, United States of America; 2 Department of Pathobiology and Veterinary Science, University of Connecticut, Storrs, Connecticut, United States of America; 3 Office of Protected Resources, NMFS, NOAA, Silver Spring, Maryland, United States of America; 4 Chicago Zoological Society’s Sarasota Dolphin Research Program, c/o Mote Marine Laboratory, Sarasota, Florida, United States of America; Animal Health Centre, CANADA

## Abstract

Following the 2010 *Deepwater Horizon* disaster and subsequent unusual mortality event, adverse health impacts have been reported in bottlenose dolphins in Barataria Bay, LA including impaired stress response and reproductive, pulmonary, cardiac, and immune function. These conditions were primarily diagnosed through hands-on veterinary examinations and analysis of standard diagnostic panels. In human and veterinary medicine, gene expression profiling has been used to identify molecular mechanisms underlying toxic responses and disease states. Identification of molecular markers of exposure or disease may enable earlier detection of health effects or allow for health evaluation when the use of specialized methodologies is not feasible. To date this powerful tool has not been applied to augment the veterinary data collected concurrently during dolphin health assessments. This study examined transcriptomic profiles of blood from 76 dolphins sampled in health assessments during 2013–2018 in the waters near Barataria Bay, LA and Sarasota Bay, FL. Gene expression was analyzed in conjunction with the substantial suite of health data collected using principal component analysis, differential expression testing, over-representation analysis, and weighted gene co-expression network analysis. Broadly, transcript profiles of Barataria Bay dolphins indicated a shift in immune response, cytoskeletal alterations, and mitochondrial dysfunction, most pronounced in dolphins likely exposed to *Deepwater Horizon* oiling. While gene expression profiles in Barataria Bay dolphins were altered compared to Sarasota Bay for all years, profiles from 2013 exhibited the greatest alteration in gene expression. Differentially expressed transcripts included genes involved in immunity, inflammation, reproductive failure, and lung or cardiac dysfunction, all of which have been documented in dolphins from Barataria Bay following the *Deepwater Horizon* oil spill. The genes and pathways identified in this study may, with additional research and validation, prove useful as molecular markers of exposure or disease to assist wildlife veterinarians in evaluating the health of dolphins and other cetaceans.

## Introduction

The northern Gulf of Mexico (nGoM) is home to 31 stocks of common bottlenose dolphins (*Tursiops truncatus*), hereafter referred to as dolphins, that inhabit bays, sounds, and estuaries (BSE) [1]. BSE dolphins are long-lived, year-round residents [e.g., 2, 3], and top predators in these coastal environments where they are routinely exposed to ecosystem perturbations, both natural [e.g., 4, 5] and anthropogenic [e.g., 6, 7]. As such, they are considered sentinels for ecosystem health. Over the past several decades, methods have been developed and refined to temporarily capture and conduct veterinary examinations to assess the health of BSE dolphins using standard diagnostic panels, including blood chemistry, complete blood cell counts, stress and reproductive hormone levels, as well as standardized physical examination and ultrasound [8–14]. The temporary capture also provides an opportunity for sampling of tissues (e.g., blood, blubber, urine) to determine exposure to chemical pollutants, pathogens, and marine toxins, supporting epidemiological studies to understand important factors affecting population health [11, 12, 15]. In the years following the explosion of the *Deepwater Horizon* (DWH) drilling platform and the subsequent release of millions of barrels of oil into the nGoM and unusual mortality event (UME, 2010–2014), wildlife veterinarians and researchers have used the aforementioned tools to identify disease conditions, many chronic, in dolphin populations exposed to the DWH oil [reviewed in 16]. Conditions have included lung disease, inflammation, impaired stress response, altered immune status, and reproductive failure [10, 11, 17–19 and others]. While great strides have been made in improving existing methods and techniques for health assessment, as well as developing new tools over the past decade, identification of additional markers of health and toxic responses would further inform health assessment and may be able to provide critical health information when full veterinary assessment is not possible.

Transcriptomic analysis is a robust tool to examine the molecular alterations that underlie toxic responses and disease and has been broadly applied in human and veterinary medicine [20, 21]. Alterations in gene expression can be extremely sensitive markers, often measurable earlier than many physical manifestations of disease or exposure. Transcriptional changes have been associated with diverse health and disease states including lung disease [22, 23] and adrenal dysfunction [24, 25] in humans and mice, cardiac disease in humans, dogs and fish [26–29], and reproductive failures in humans [30]. Blood plays a critical role in immunity and physiological homeostasis and, due to the recirculation of immune cells between the blood and lymphoid tissues or migration to sites of pathological insult, contains a broad range of transcripts. These transcripts can be indicative of pathological changes in other organs of the body or systemic exposures and have been successfully applied in humans and other companion or economically relevant species [reviewed in 20, 21]. However, application in wildlife species has been more limited due in part to the logistical challenges in sample collection and limited molecular resources for many species. Nonetheless, blood gene expression profiles have been investigated in multiple marine mammals including the beluga whale (*Delphinapterus leucas*) [31], killer whale (*Orcinus orca*) [32], harbor porpoise (*Phocoena phocoena*) [33], Indo-Pacific humpback dolphin [34] and grey seal (*Halichoerus grypus*) [35] and associated with disease state and/or nutritional status in Steller (*Eumetopia jubatus*) and California (*Zalophus californianus*) sea lions [36, 37], polycyclic aromatic hydrocarbon (PAH) exposure in the sea otter (*Enhydra lutris*) [38], and stress response, polychlorinated biphenyl (PCB) exposure, sex, geography and seasonality in the common bottlenose dolphin [39–42].

Previous analyses of the skin transcriptome of dolphins living in significantly oiled areas of the nGoM did not identify overexpression of transcripts in classical detoxification pathways of oil (i.e., involving cytochrome P450s and the aryl hydrocarbon receptor) [43, 44]. Changes in cytoskeletal, immune, and oxidative transcripts were observed, but were strongly associated with seasonal differences, making it difficult to detect any potential correlation to oil exposure [43, 44]. However, these pathways have been associated with the molecular response to oil in other species [e.g., 26, 27, 45–47] and to other contaminants of emerging concern in bottlenose dolphins [48]. The substantial influence of the external environment (e.g., water/air temperature) on the skin transcriptome is not surprising and it is reasonable to assume that internal tissues (e.g., blood) would be less influenced because of physiological homeostasis. In fact, blood transcriptomes from managed-care dolphins have identified a broader suite of expressed transcripts with more limited seasonal effects [41], suggesting that gene expression profiling of blood may be a more powerful tool to assess the health impacts from exposure to oil. Morey et al. [41] also correlated gene expression in blood to standard hematological parameters allowing for interpretation of the molecular data in the broader scope of animal health.

The current study leverages the broad portfolio of health data accumulated from dolphin health assessments during 2013–2018 in areas that were heavily oiled following the DWH disaster, Barataria Bay, LA (BAR), and non-oiled waters of Sarasota Bay, FL (SAR) [49]. Both populations have been well studied, SAR for five decades and BAR for the 10 years following DWH [e.g., 9, 10, 13, 17, 18, 50–52]. Transcriptome profiles of blood were compared to concurrently collected biochemical and physiological measurements and observed disease states with the goal to identify additional pathways and molecular markers that may be further investigated and developed to assist researchers and wildlife veterinarians in assessing the health status of dolphins.

## Materials and methods

### Sample collection

**Fig 1:**
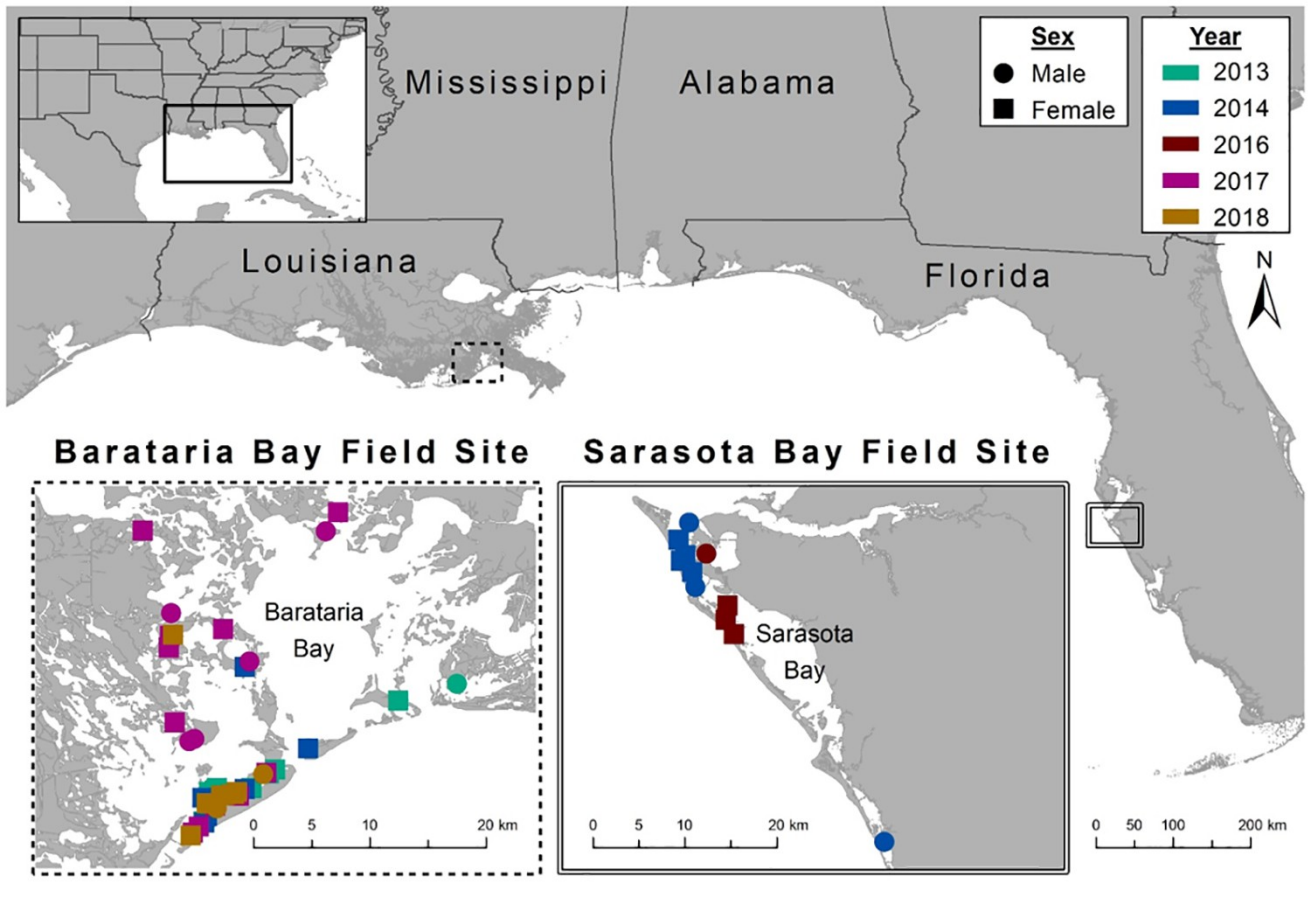
Capture-release health assessment locations for dolphins sampled for blood transcriptomic analysis. The image was created by the authors in ESRI’s ArcGIS 10.1, no copyrighted material was used.

Dolphin capture-release health assessments were conducted in and around the waters of Barataria Bay, LA during June 2013, June 2014, June and September 2017, and July 2018 and Sarasota Bay, FL during May 2014 and 2016 (Fig 1). Dolphin handling followed previously described protocols [13]. Briefly, a group of 1–4 dolphins was encircled by a seine net and safely restrained by experienced handlers for processing in the water and/or on board a vessel. Diagnostic sampling procedures for a suite of health measures have been described elsewhere [11, 18]. Blood for transcriptomic analyses was drawn from the periarterial venous rete accessed from the ventral aspect of the fluke and collected into a PAXGene blood RNA tube (Qiagen, Valencia, CA). The blood was mixed well and subjected to step-down freezing prior to cryogenic storage. Any BAR dolphins with observational life history established by photographic-identification as a neonate or young of year were assigned to pre- or post-DWH oil spill birth cohorts. The samples selected represented diversity in age class, birth cohort, sex, pregnancy status, reproductive outcome, and health panels as defined in [11] (Table 1 and S1 Table).

**Table 1:** Samples analyzed by RNA-seq.

Field ID	Collection Date	Location	Length (cm)	Sex	Pregnant	Birth cohort
Y26	6/17/2013	BAR	196	Male		pre-spill
Y30	6/18/2013	BAR	192	Male		post-spill
Y41	6/18/2013	BAR	223	Female	Yes	pre-spill
Y43	6/18/2013	BAR	242	Female	No	pre-spill
Y45	6/19/2013	BAR	227	Female	No	pre-spill
Y47	6/21/2013	BAR	228	Female	No	pre-spill
Y49	6/21/2013	BAR	221	Female	No	pre-spill
Y57	6/23/2013	BAR	208	Female	No	pre-spill
Y44	6/25/2013	BAR	221	Male		pre-spill
Y06	6/26/2013	BAR	225	Male		pre-spill
Y63	6/26/2013	BAR	236	Female	Yes	pre-spill
Y65	6/27/2013	BAR	236	Female	Yes	pre-spill
Y50	6/10/2014	BAR	204	Male		post-spill
Y26	6/12/2014	BAR	202	Male		pre-spill
Y34	6/12/2014	BAR	215	Male		pre-spill
Y83	6/16/2014	BAR	243	Female	Yes	pre-spill
Y85	6/16/2014	BAR	230	Female	Yes	pre-spill
Y91	6/17/2014	BAR	243	Female	Yes	pre-spill
Y15	6/18/2014	BAR	210	Female	No	pre-spill
Y56	6/18/2014	BAR	275	Male		pre-spill
Y95	6/18/2014	BAR	238	Female	No	pre-spill
Y99	6/19/2014	BAR	259	Female	Yes	pre-spill
YA1	6/19/2014	BAR	251	Female	Yes	pre-spill
YA3	6/19/2014	BAR	241	Female	Yes	pre-spill
YK9	6/14/2017	BAR	214	Female	No	CBD
Y92	6/15/2017	BAR	231	Male		pre-spill
YN1	6/15/2017	BAR	186	Female	CBD^1^	post-spill
YN3	6/15/2017	BAR	193	Female	CBD	post-spill
Y94	6/16/2017	BAR	213	Male		post-spill
YN5	6/16/2017	BAR	214	Female	No	post-spill
YN9	6/17/2017	BAR	222	Female	Yes	pre-spill
YR1	6/17/2017	BAR	197	Female	CBD	post-spill
Y96	6/19/2017	BAR	185	Male		CBD
YR3	6/19/2017	BAR	234	Female	No	CBD
Y98	6/23/2017	BAR	256	Male		pre-spill
YR5	6/23/2017	BAR	197	Female	CBD	post-spill
YV5	9/18/2017	BAR	245	Female	No	pre-spill
Y19	9/19/2017	BAR	241	Female	Yes	pre-spill
YA2	9/19/2017	BAR	248	Male		pre-spill
YA3	9/19/2017	BAR	237	Female	Yes	pre-spill
YV7	9/19/2017	BAR	226	Female	No	pre-spill
YA6	9/20/2017	BAR	258	Male		pre-spill
YX1	9/20/2017	BAR	245	Female	No	pre-spill
YX3	9/20/2017	BAR	238	Female	No	pre-spill
Y99	9/21/2017	BAR	256	Female	Yes	pre-spill
YF0	9/21/2017	BAR	232	Male		pre-spill
YF4	9/21/2017	BAR	203	Male		post-spill
YX5	9/21/2017	BAR	245	Female	Yes	pre-spill
Y93	7/10/2018	BAR	235	Female	Yes	pre-spill
YF8	7/10/2018	BAR	258	Male		pre-spill
YX7	7/10/2018	BAR	250	Female	Yes	pre-spill
Y66	7/11/2018	BAR	220	Male		CBD
Y79	7/11/2018	BAR	232	Female	Yes	pre-spill
YJ2	7/12/2018	BAR	254	Male		pre-spill
YX9	7/12/2018	BAR	234	Female	Yes	CBD
YV7	7/13/2018	BAR	233	Female	Yes	pre-spill
YA6	7/16/2018	BAR	262	Male		pre-spill
YY3	7/16/2018	BAR	235	Female	No	pre-spill
YY9	7/19/2018	BAR	243	Female	Yes	pre-spill
YZ1	7/20/2018	BAR	259	Female	CBD	pre-spill
196	5/5/2014	SAR	269	Male		
268	5/5/2014	SAR	273	Male		
284	5/5/2014	SAR	211.5	Male		
276	5/6/2014	SAR	285	Male		
197	5/7/2014	SAR		Female	Yes	
133	5/8/2014	SAR	242	Female	No	
175	5/8/2014	SAR	249	Female	CBD	
249	5/9/2014	SAR	203	Female	No	
209	5/6/2016	SAR	236	Female	No	
255	5/6/2016	SAR	192	Female	CBD	
33	5/7/2016	SAR	258	Female	No	
292	5/9/2016	SAR	216	Male		
178	5/11/2016	SAR	272	Male		
188	5/11/2016	SAR	257	Male		
223	5/11/2016	SAR	251	Female	No	
294	5/11/2016	SAR	202	Male		

^1^CBD: Cannot be determined

### RNA extraction and sequencing

Whole blood RNA was extracted per manufacturer’s protocol using the PAXGene Blood RNA kit (Qiagen, Valencia, CA) with on-column DNase digestion. RNA was quantified with a NanoDrop 1000 (Thermo Fisher Scientific, Wilmington, DE) and Qubit (Invitrogen) and quality was assessed with a Bioanalyzer 2100 (Agilent Technologies, Inc., Santa Clara, CA). Only samples with an RNA Integrity Number ≥ 7 were sequenced. Total RNA from blood (n = 76) was sent to North Carolina State University’s Genomics Sciences Laboratory for library preparation using the NEBNext Ultra Directional RNA Library Prep Kit for Illumina with NEBNext Multiplex Oligos for Illumina (New England BioLabs, Ipswich, MA). Sequencing was performed on an Illumina NovaSeq 6000, generating 150 base paired-end reads to a targeted depth of at least 50M reads per sample.

### Sequence processing

Sequence processing and analysis were carried out in CyVerse’s Discovery Environment using the high-performance computing applications [53, 54]. Trimmomatic [55] was used to groom raw sequences and quality was checked before and after grooming with FastQC [56] (HTProcess-prepare_directories-and-run_fastqc-0.1 and HTProcess_trimmomatic_0.33 apps; phred score > 20). Reads were mapped to the Tur_tru v1 genome (RefSeq accession GCF_001922835.1) with Tophat2 [57] using Bowtie2 [58] (HTProcess_Tophat-2.1.1 app). Cufflinks [59] generated FPKM (fragments per kilobase of transcript per million mapped reads) counts that were used for all additional analyses (HTProcess_Cufflinks-2.2.1 app). Due to their abundance in the blood transcriptome, hemoglobin sequences HBB, HBM, and HBZ were masked from the genome and excluded from further analyses.

### Expression analyses

Data were filtered prior to further analyses to establish sets of “expressed” transcripts including only transcripts with a mean FPKM ≥ 1 across all samples and FPKM > 0 in at least half of the samples. Principal component analysis (PCA) and weighted gene co-expression network analysis (WGCNA) were performed in RStudio v1.1.463 [60] running R v 3.5.2 [61]. PCA was conducted with the FactoMineR [62] and factoextra [63] packages and WGCNA [64] was used for network analysis. Differential expression analyses were conducted with Cuffdiff [59] in CyVerse’s Discovery Environment. Over-representation analysis (ORA) and pathway mapping of differentially expressed gene sets or modules of co-expressed genes, compared to their respective background gene set, was completed in WebGestalt using annotated gene symbols [65]. Statistical significance from Cuffdiff and WebGestalt required a Benjamini and Hochberg adjusted p-value ≤ 0.05. All analyses used data mapped to the gene level of annotation.

## Results and discussion

### Dolphin blood transcriptome

**Fig 2:**
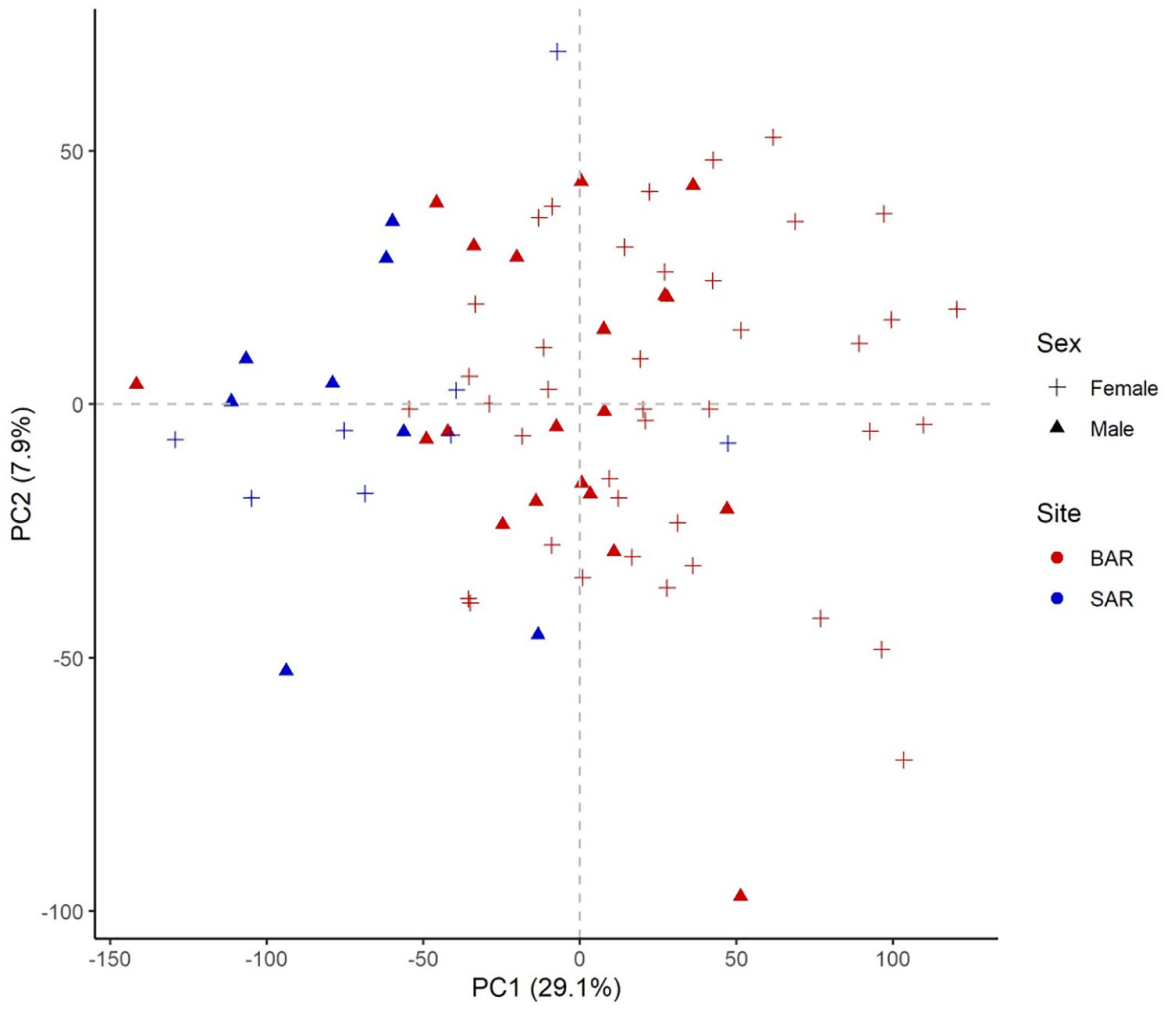
Principal component analysis with 11,599 expressed genes identified in the blood transcriptome of dolphins from Barataria Bay, LA (BAR) and Sarasota Bay, FL (SAR). Principal component 1 accounted for 29.1% of variability and PC2 for an additional 7.9%.

**Fig 3:**
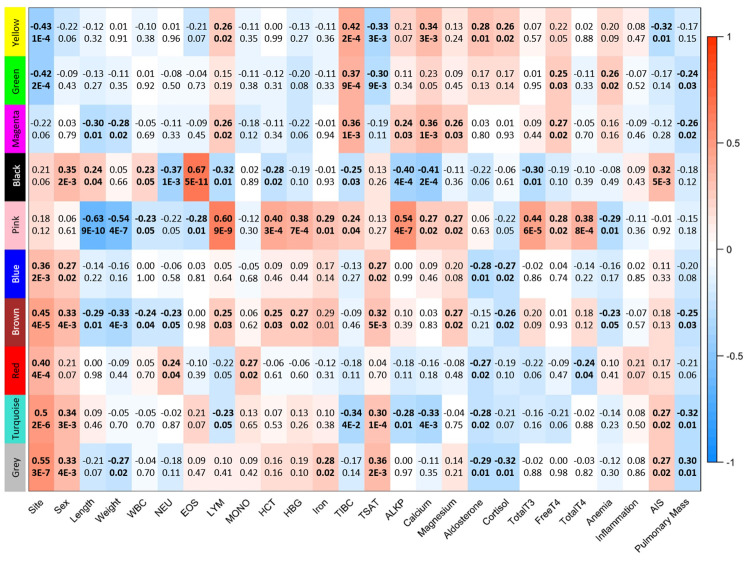
WGCNA module-trait relationships from analysis of 76 dolphin blood transcriptomes from Barataria Bay, LA and Sarasota Bay, FL. Nine modules of co-expressed genes were identified; the grey module contains all genes that were not assigned to any co-expression module. For selected traits, correlation coefficients and associated p-values are shown, significant correlations are bolded. WBC: white blood cells, NEU: % neutrophils, EOS: % eosinophils, LYM: % lymphocytes, MONO: % monocytes, HCT: hematocrit, HBG: hemoglobin, TIBC: total iron binding capacity, TSTAT: saturation of transferrin, TotalT3: total triiodothyronine, TotalT4: total thyroxine, AIS: alveolar-interstitial syndrome.

Blood transcriptomes from 76 samples (60 from BAR and 16 from SAR) collected from 71 unique animals during 2013–2018 were analyzed (Table 1, S1 Table, and Fig 1). After filtering of the comprehensive dataset (mean FPKM ≥ 1 and FPKM > 0 in at least 38 samples), we defined 11,599 genes as “expressed” in our blood transcriptome, representing 48% of genes in the Tur_tru v1 genome assembly. PCA did not reveal a distinct separation of samples based on gene expression (Fig 2). It is unlikely that sex-specific gene expression would confound analysis and interpretation as only 22 genes (0.19%) exhibited differential expression between males and females. WGCNA identified nine modules of co-expressed genes, including 4,153 genes (36%), many of which were correlated to health parameters measured during health assessments (Fig 3, S2 Table).

### Variation between BAR and SAR transcriptional profiles

The PCA showed a shift in expression profiles between BAR and SAR on PC1 (Fig 2), with the majority of transcripts more highly expressed in BAR. However, only 154 genes (1.3%) exhibited differential expression between BAR and SAR of which the majority (90%) were more highly expressed in SAR (S3 Table). This differential expression is substantially less than previously reported for dolphin skin transcriptomes (23.5%), but in addition to the difference in tissue, the Neely et al. study encompassed a greater geographic region, comparing animals in the nGoM with dolphins from the southeastern US (SE US) Atlantic [43]. WGCNA further demonstrated the influence of location on blood gene expression with 6 of 9 co-expressed gene modules exhibiting correlation to site (Fig 3). The brown (247 genes, r = 0.45, p = 3.72e-5) and blue (423 genes, r = 0.36, p = 1.64e-3) modules consist of genes generally expressed at higher levels in BAR. Pathways and GO terms associated with oxidative phosphorylation (OXPHO) and/or the mitochondria dominated the results for the blue module. The other four modules mapped with similarities to observations among the much smaller set of differentially expressed genes as discussed below.

The majority of annotated genes differentially expressed between sites were ribosomal-associated genes, resulting in over-representation of pathways and GO terms associated with ribosomes and translation. This trend was also observed in WGCNA analyses where the green module exhibited elevated transcript expression in SAR (86 genes, r = -0.42, p = 1.61e-4) and was primarily comprised of genes associated with the ribosome and translation (Fig 3). It is interesting to note that a previous study of skin transcriptomes of male dolphins from the nGoM, including BAR but not SAR, and the SE US Atlantic coast identified increased expression of ribosomal-associated transcripts in nGoM animals, relative to the SE US coast [43]. Transcriptional regulation of ribosomal genes is a crucial but complex process in mammals and the underlying cause of this differential expression cannot be adequately addressed by these studies; however, down-regulation has been associated with oil exposure in fish [27]. The observed disparities in ribosomal transcript expression between BAR and SAR could be attributed to anything from basic physiological differences in growth rates or nutritional status to nucleolar stress to epigenetic modifications induced by PAHs or a host of other stressors.

The yellow module (153 genes, r = -0.43, p = 1.26e-4) also consists of genes that were more highly expressed in SAR (Fig 3). This module is enriched in genes mapping to ferroptosis and heme biosynthesis pathways and GO terms including iron ion binding, metal ion homeostasis, and oxidoreductase activity. Correspondingly, gene expression in this module was also positively correlated to TIBC (r = 0.42, p = 1.82e-4) and negatively correlated to saturation of transferrin (r = 0.33, p = 3.23e-3) serum measurements. Alterations in expression of these genes could be associated with the reduction of oxygen transport due to oil exposure, an observation well documented in fish and tied to reduction in β-hemoglobin transcripts that persist even in the absence of oil [45 and references therein]. β-hemoglobin transcripts were masked from this analysis, but data from unmasked sequence alignments indicates that it was down-regulated approximately 1.6 fold in BAR (FPKM_SAR_ = 1,314,283; FPKM_BAR_ = 917,973). Thus, it is possible that oil-induced reductions in oxygen transport are observed in BAR dolphins, either due to chronic, low-level PAH exposure or associated with compromised health still observed years after the DWH spill [18, 19].

There was indication of increased immune activity in BAR with 9 of the 16 genes more highly expressed in BAR involved in anti-viral or other immune responses. This was further supported by over-representation of immune response genes identified in the red (86 genes, r = 0.40, p = 3.42e-4) and turquoise (2948 genes, r = 0.5, p = 1.78e-6) modules of co-expressed genes. Similar to the differential expression results, the genes in these modules were found to be more highly expressed in animals from BAR, compared to SAR, and expression profiles were more highly correlated to site than other common immune indicators including WBC differentials (Fig 3). Down-regulation of immune transcripts in response to oil, in contrast to up-regulation observed in BAR dolphins, has been reported for several fish species; however, extended oil exposure shifted trends to up-regulation [46]. Further, the up-regulation of immune transcripts observed in BAR may correspond to an increase in T lymphocyte proliferation observed in BAR dolphins sampled in 2011 [17] and thereafter [19], compared to SAR. The BAR samples included in this study were collected 3+ years following the DWH spill, but it is possible that the up-regulation of immune transcripts may be associated with chronic exposure from DWH oil (e.g., sustained surface slicks, buried oil) or continuing oil industry in BAR.

Evidence of a Th1 response indicative of a reaction to intracellular pathogens was largely absent as transcripts with homology to interferon-γ (IFNG) or its receptor were not expressed in blood transcriptomes of this study. This may be correlated with observations of reduced serum Th1 cytokines, Il-2, IL-12, and INFG, in BAR dolphins sampled in 2011–2018, relative to SAR [17, 19]. Interestingly, the red module did show over-representation of genes mapping to the *Type II Interferon Signaling (IFNG)* WikiPathway (p = 8.14e-7). However, closer examination revealed that the genes involved are well downstream of the IFNG/IFNGR complex and many are more broadly involved in cytokine responses via JAK/STAT signaling, with some also mapping to IL-4 and IL-6 signaling pathways. This supports evidence of an elevated Th2 gene expression response in BAR, with over-representation of upregulated transcripts in the turquoise module involved in *IL-4 Signaling* (p = 5.96e-5), *IL-5 Signaling* (p = 6.68e-4) and *IL-6 Signaling* (p = 9.10e-5) WikiPathways, largely among components of JAK/STAT signaling. The molecular-based immune response described here is likely reflected in standard hematology panels, as the red module correlated with increasing neutrophil differentials (r = 0.24, p = 0.04) and the turquoise module with decreasing lymphocyte differentials (r = -0.23, p = 0.05, Fig 3).

Additionally, IL-4 like cytokine-cytokine receptor interactions and IL-17E, also known as IL-25, signaling were well represented in the turquoise module when mapped to KEGG pathways. IL-25 is known to regulate the Th2 response against helminthic parasites [66], a prevalent intestinal fauna in *T*. *truncatus* [67]. Transcripts for IL-10 were expressed 2 fold higher in BAR than in SAR and increased transcript expression was also observed for IL-10 receptors. Interestingly, serum concentrations of IL-10A were lower in BAR dolphins than in SAR [19]; however it is important to note that the relationship between transcript and protein expression is highly complex. Overall, gene expression profiles are similar to increases in serum Th2 cytokines in BAR dolphins, relative to SAR, that have been identified post-DWH (2011–2018) and support findings that BAR mononuclear cells were more responsive to Th2 stimulation than were SAR dolphin cells [17, 19]. The alterations in Th2 transcript profiles between BAR and SAR dolphins may be influenced by oil exposure, as *in vitro* exposure of SAR dolphin mononuclear cells exhibited increased responsiveness to Th2 stimulation [19].

Evidence of a Th17 response is also observed in the turquoise module with TGFβ influenced Th17 differentiation well mapped in C*ytokine-Cytokine Receptor Interactions*, *Th17 Cell Differentiation* and *IL-17 Signaling* KEGG pathways and *IL-17 Signaling* (p = 1.84e-3) WikiPathway. In particular, IL-17A and IL-17F signaling, with roles in acute and chronic inflammatory responses, are well represented. While inflammation is well documented among the BAR dolphin population [11, 18], no significant correlation between inflammation and the turquoise WGCNA module was observed (Fig 3). However, the red module, which contains up-regulated genes mapping to GO biological process terms including *Response to Type I Interferon* (p = 2.44e-11), *Regulation of Response to Cytokine Stimulus* (p = 6.8e-6), and *STAT Cascade* (p = 4.9e-4), is correlated with increasing monocyte differentials (r = 0.27, p = 0.02) and inflammation (r = 0.21, p = 0.07, Fig 3). TGFβ is also important in the regulation and function of regulatory T (Treg) cells, which were more abundant in circulation in BAR than SAR dolphins in the absence of increased Treg cytokines, suggesting some dysregulation of Treg cells [19].

The cardiotoxic effects of oil have been well documented in several species including fish, birds, and humans [e.g., 26, 68, 69]. Echocardiography is difficult in living cetaceans, although it has been successfully applied in both wild and managed-care dolphins [70], therefore additional markers of cardiovascular status would be useful for health assessment. The turquoise WGCNA module resulted in over-representation of genes mapping to WikiPathways *Hypothesized Pathways in Pathogenesis of Cardiovascular Disease* (p = 0.04) and *Cardiac Hypertrophic Response* (p = 6.61e-4) and was correlated with decreasing serum calcium concentrations (r = -0.33, p = 3.52e-3, Fig 3). All components of the transforming growth factor beta complex are upregulated in BAR, which, once activated, plays a role in development of cardiovascular disease, regulating endocardial and epicardial epithelial-to-mesenchymal transition, neural crest migration, extracellular matrix remodeling, cell differentiation and development, and maintenance of cardiovascular structure and function via canonical and non-canonical pathways [reviewed in 29]. The correlation of this module to calcium is of note as dysregulation of calcium signaling has been shown to play a role in oil- and/or dispersant-associated cardiac effects [e.g., 71]. Several genes were proposed as molecular markers of oil-induced cardiotoxicity in fish, and while the piscine suite of genes does not overlap with those annotated in the pathways discussed here, cytoskeletal and contractile functions dominate, indicating similar endpoints for cardiotoxicity in both fish and dolphins [26]. Identification of molecular markers of cardiovascular disease in the dolphin blood transcriptome may, with further research, lead to development of sensitive indicators of cardiac abnormalities that can further inform health assessment in the absence of echocardiography.

Over-representation of the KEGG pathways *Non-Small Cell Lung Cancer* (p = 4.25e-4) and *Small Cell Lung Cancer* (p = 4.38e-3) was observed in the turquoise WGNA module and was positively correlated with alveolar-interstitial syndrome (AIS, r = 0.27, p = 0.02, Fig 3). Lung cancer has not been reported in BAR dolphins, but lung disease and worsening AIS has been reported [11, 18]. It is likely that the aforementioned KEGG pathways function more generally in lung disease through their roles in cell cycle, proliferation, and apoptotic control. In fact, the over-expression of genes in cancer pathways, more so than inflammatory or immune pathways, has been linked to chronic fibrosing idiopathic interstitial pneumonia in humans [72]. The genes mapped in the KEGG pathway are primarily involved in PIK3/AKT, MAPK, Erb, or RAS signaling. There is significant crosstalk between these pathways, ultimately leading to uncontrolled cellular proliferation and anti-apoptosis, both characteristic of lung fibrosis. Additionally, chronic AKT expression has been found to predispose rodents to fibrosis of lung tissue [73, 74]. Both AKT1 and AKT2 were expressed at higher levels in BAR and may be an indicator of the molecular mechanisms underlying the increased lung fibrosis observed in BAR dolphins. With further research, the genes identified in these lung cancer pathways may be developed as molecular indicators of lung disease in the dolphin blood transcriptome.

### Alterations in BAR transcript profiles over time

**Fig 4:**
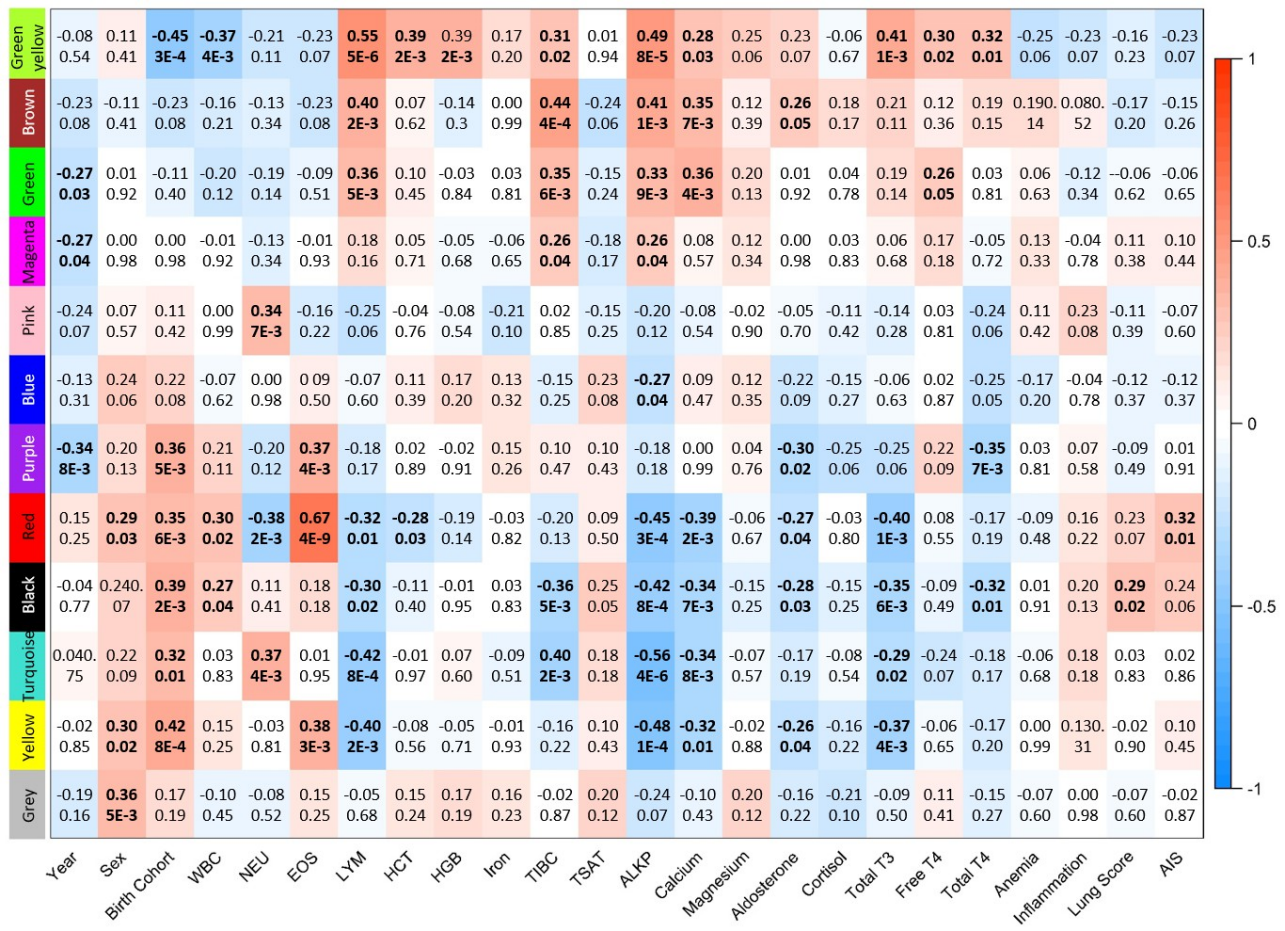
WGCNA module-trait relationships from analysis of dolphin blood transcriptomes from Barataria Bay, LA. Eleven modules of co-expressed genes were identified; the grey module contains gene that were not assigned to any co-expression module. For selected traits correlation coefficients and associated p-values are shown, significant correlations are bolded. WBC: white blood cells, NEU: % neutrophils, EOS: % eosinophils, LYM: % lymphocytes, HCT: hematocrit, HBG: hemoglobin, TIBC: total iron binding capacity, TSTAT: saturation of transferrin, ALKP: alkaline phosphatase, Total T3: total triiodothyronine, Free T4: free thyroxine, Total T4: total thyroxine, AIS: alveolar-interstitial syndrome.

To further examine the relationship between blood transcript expression and adverse health outcomes observed in BAR dolphins, the 60 samples (20 male, 40 female, Table 1) collected from BAR were analyzed independently of SAR samples. Blood samples span a six-year period, providing a longitudinal study of the BAR dolphin population; however, the earliest samples, from 2013, were collected three years post-DWH oiling. After filtering (mean FPKM ≥ 1 and FPKM > 0 in at least 30 samples), 11,581 genes were defined as “expressed”, similar to the previous analysis that included SAR samples. WGCNA organized 2,382 genes (21%) into 11 co-expression modules with significant correlation to various health parameters (Fig 4, S4 Table). Again, minimal differential expression was observed between sexes, with only seven genes reporting expression differences between males and females.

**Fig 5:**
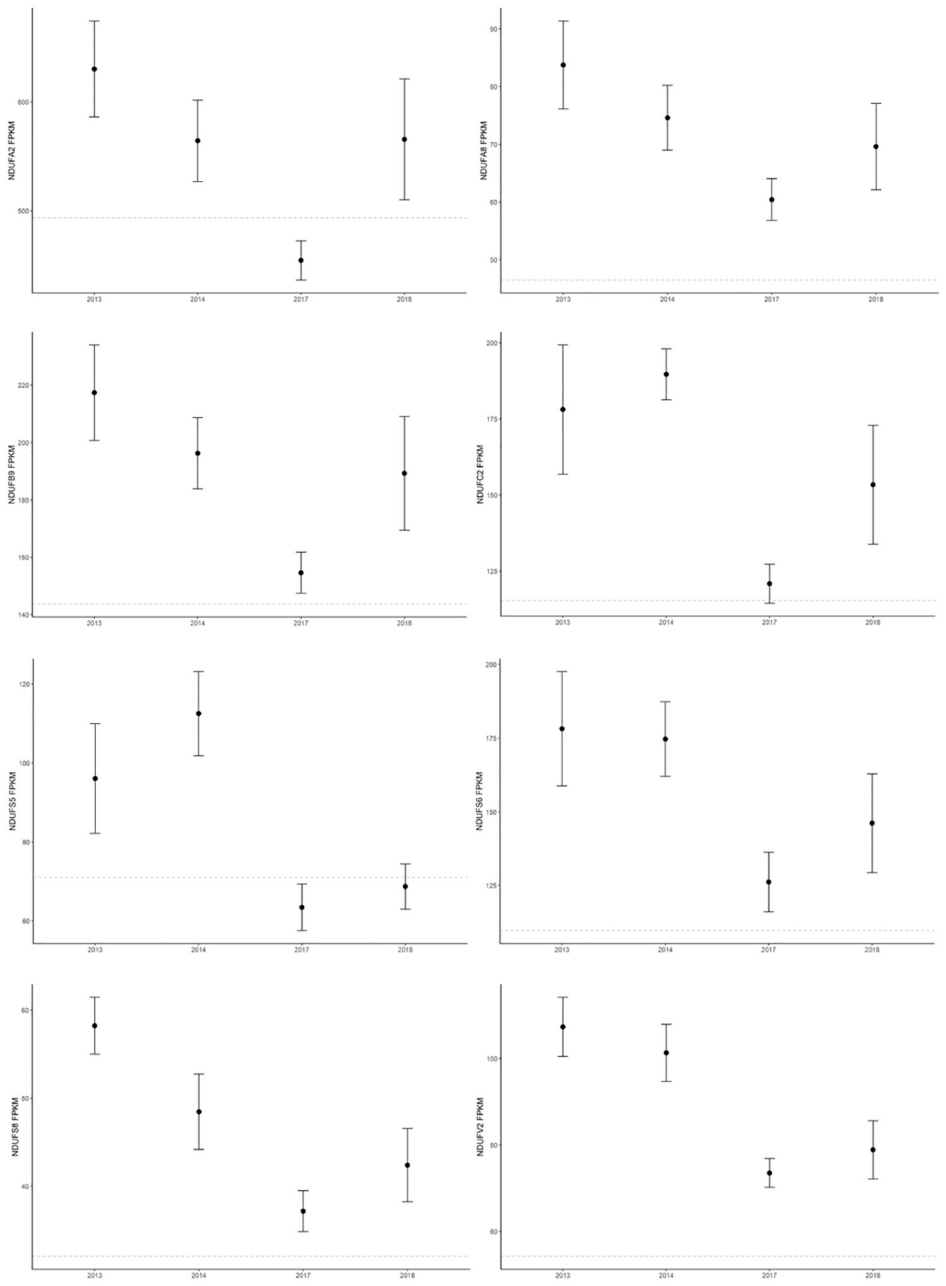
Expression of OXPHO Complex I genes in BAR over time (mean ± SE). Dashed line is the mean expression in SAR.

There was differential expression of 103 genes (87 unique) when analyzed as a time course (S5 Table) or 265 genes (180 unique) between years for all pairwise comparisons (S6 Table). Gene expression profiles in 2013, the first year sampled post-DWH, were the primary source of difference between years; however, 2017 also exhibited substantial differences from other years. Over-representation of GO terms associated with immune response were identified. However, KEGG and WikiPathway analysis instead revealed over-representation of genes involved in the *Electron Transport Chain (OXPHOS System in Mitochondria)* (p = 3.72e-7) and *Oxidative Phosphorylation* (p < 5.56e-5) pathways. In particular, 25% of the annotated genes encoding proteins for Complex I (NADH-ubiquinone oxidoreductase) in the dolphin blood transcriptome are differentially expressed. Overall, expression of these genes was highest in 2013 and/or 2014, decreased substantially by 2017, followed by a slight increase in 2018, and is generally higher than measured in SAR dolphins (Fig 5).

Complex I is a critical component of mammalian cellular respiration and metabolism that can be disrupted by environmental, pathological, or genetic factors [reviewed in 75]. Dysfunction in Complex I results in impaired catalytic ability and increased superoxide production that have widespread consequences in health including mitochondrial disease, neurodegenerative disease, apoptotic dysfunction, reproductive dysfunction, and impacts on brain, heart, or skeletal muscle [reviewed in 76]. As such, over-representation of transcripts mapping to *Parkinson’s Disease* (p = 3.88e-7), *Alzheimer’s Disease* (p = 6.13e-5), *Huntington’s Disease* (p = 3.51e-5), N*on-Alcoholic Fatty Liver Disease* (p = 3.46e-4), and *Thermogenesis* (p = 3.26e-4) KEGG pathways resulted from perturbations in OXPHO. Similarly, ORA of genes more highly expressed in the skin of nGoM dolphins than SE US dolphins significantly mapped to these pathways in an earlier study [43]. The disruption of OXPHO due to crude oil and other PAHs is well documented [77, 78] and the altered expression observed in 2013 and 2014 may be associated with oiling from the DWH disaster. Heritable alterations in oxidative phosphorylation have been observed in killifish chronically exposed to PAHs [78], thus the sustained elevated expression, relative to SAR, may be baseline for BAR dolphins who likely encounter chronic low-level PAH exposure due to seeps and small-scale oil spills associated with industry in the BAR region.

To more directly address changes that may be attributed to substantial oil exposure following the DWH disaster, any dolphins with observational life history established by photographic-identification as a neonate or young of year were assigned to pre- or post-DWH oil spill birth cohorts. One hundred one genes exhibited differential expression between animals known to be alive at the time of the DWH spill and those known to be born after the spill (S7 Table); however, there was no significant over-representation among these genes. Of note is PRG3, expressed 8.2 fold higher in the pre-spill birth cohort. PRG3 is a cytoplasmic protein with putative oxidoreductase activity responsive to mitochondrial-released apoptosis inducing factors, resulting in caspase-independent apoptosis [79]. Upregulation of PRG3 transcript expression has been observed in human studies following acute (≤ 24 h) exposure to benzo[α]pyrene and TCDD, although to a much lesser extent, 1.72 and 2.02 fold, respectively [80]. Additionally, prolonged exposure to cigarette smoke condensates (9 months) resulted in epigenetic modifications (hypermethylation) of PRG3 in human lung cancer cell lines [81]. Thus, the substantial upregulation of PRG3 transcripts observed 3+ years post-spill in BAR dolphins likely exposed to sustained crude oil slicks following the DWH explosion may be associated with oil-induced epigenetic modifications. With further research, PRG3 may be useful as a molecular marker of sustained PAH exposure in dolphins.

Six WGCNA modules exhibited correlation to birth cohort (Fig 4). The greenyellow module consisted of 35 genes expressed more highly in the cohort born post-spill (r = -0.45, p = 2.68e-4, Fig 4). Despite the limited number of genes in the module, over-representation of genes mapping to the KEGG pathway *Hematopoietic Cell Lineage* (p = 0.005) was observed and appears to indicate increased B cell differentiation/proliferation among the post-spill birth cohort with CD20, CD21, and CD22 expressed more highly in these dolphins. Similarly, genes of the greenyellow module mapped to the *B Cell Receptor Signaling* WikiPathway (p = 0.007), including expression of CD79B. B cell differentiation and proliferation can be influenced by any number of biotic or abiotic factors and interactions can be complex. For example, *in vitro* dolphin studies reported an increase in lymphocyte proliferation following acute crude oil exposure but a decrease when exposed to mixtures of crude oil and chemical dispersants [82]. Thus, we cannot determine the cause of decreased expression of transcripts involved in B cell proliferation among the pre-spill birth cohort from this study.

All other modules consisted of genes expressed more highly in animals born pre-spill, thus likely to have encountered surface slicks of oil and associated aerosolized contaminants, including the turquoise module (785 genes, r = 0.32, p = 0.01), red module (96 genes, r = 0.35, p = 5.84e-3), purple module (50 genes, r = 0.36, p = 4.69e-3), yellow module (200 genes, p = 0.42, r = 8.03e-4), and the black module (74 genes, r = 0.39, p = 1.94e-3, Fig 4). The large turquoise module primarily consisted of genes involved in immune responses. The specific pathways and GO terms mapped are similar to those previously associated with location indicating that BAR animals likely exposed to significant oiling are driving alterations in transcriptome profiles compared to the reference site. Given the earlier discussion of dysregulation of Treg cells in BAR dolphins, it is also interesting to note that, while not statistically significant, both the yellow and purple modules mapped to immune-related pathways including *T-Cell Receptor and Co-stimulatory Signaling* and *TGF-beta Signaling Pathway* WikiPathways along with the *Aryl Hydrocarbon Receptor* WikiPathway. In addition to its role in classical detoxification pathways, the aryl hydrocarbon receptor (AhR) plays a role in the control of Treg and Th17 cell differentiation [83], further supporting the potential role of oil exposure in the immune-related alterations observed in BAR dolphins.

Furthermore, the classical detoxification pathways involving cytochrome P450s and AhRs are also involved in regulation of eicosanoid synthesis [84] and the mapping to AhR pathways observed in the purple and yellow modules was due, in part, to prostaglandin expression and associated EGFR signaling pathways. Thus, mapping of genes in the red module to *Eicosanoid Synthesis* and *Prostaglandin Synthesis and Regulation* WikiPathways in ORA, driven by a small group of genes involved in the *Arachidonic acid metabolism* KEGG pathway, may also be regulated by the classical detoxification pathways. PAH exposure and the associated alterations in eicosanoids are involved in a wide array of adverse health effects including pulmonary impairment [84]. The alterations observed in eicosanoid synthesis among pre-spill birth cohort animals may indicate the role of PAH exposure associated with increased lung disease in BAR dolphins [11, 18].

Genes of the black module mapped to cytoskeletal GO terms and pathways in ORA, primarily represented by actin- and myosin-related proteins. Likewise, genes of the yellow module mapped to cytoskeletal regulation, particularly cellular junction formation. Alterations in cytoskeletal structure following PAH exposure, both at the gene and protein level, are well documented and are often secondary to PAH-induced oxidative or apoptotic responses; however, down-regulation is more commonly reported and exposure to oil dispersant can confound the relationship [e.g., 47, 85]. Similar upregulation of cytoskeletal genes associated with increasing persistent organic pollutant concentrations was observed in the skin transcriptomes of dolphins in the nGoM and the SE US [43]. More specific to PAH exposure, alterations in cytoskeletal transcripts were measured in skin collected from dolphins in the nGoM prior to or after visible oiling from the DWH spill, but due to the strong influence of season, a conclusive interpretation of PAH effects was not possible [44]. Nonetheless, the data presented here provides strong evidence for differences in structural processes between dolphins likely exposed to significant oiling vs those that were not yet born at the time of the DWH disaster.

### Transcriptional markers of pregnancy and reproductive outcome

Reproductive failure in the BAR dolphin population following the DWH oil spill is well documented, with a low success rate of approximately 20% in comparison to 83% success in SAR [86], and determining pregnancy status and outcomes has been an integral part of health assessments. Pregnancy can be established through progesterone levels measured in blood or blubber, but ultrasound is considered the definitive method to establish pregnancy status, trimester, and pregnancy viability [10]. Some hematological and biochemical markers of pregnancy outcome have been described in dolphins managed under human care; however, proper interpretation requires knowledge of trimester and, often, repeated sampling to identify changes throughout pregnancy [87]. Additionally, the alterations in these blood-based markers are most pronounced in the third trimester, whereas to date, the majority of pregnant dolphins sampled during health assessments are in the first trimester. Consequently, the blood transcriptomes of females were queried to identify transcripts associated with pregnancy or pregnancy outcome that may be an avenue for further research in identifying markers of reproductive status or outcome.

**Fig 6:**
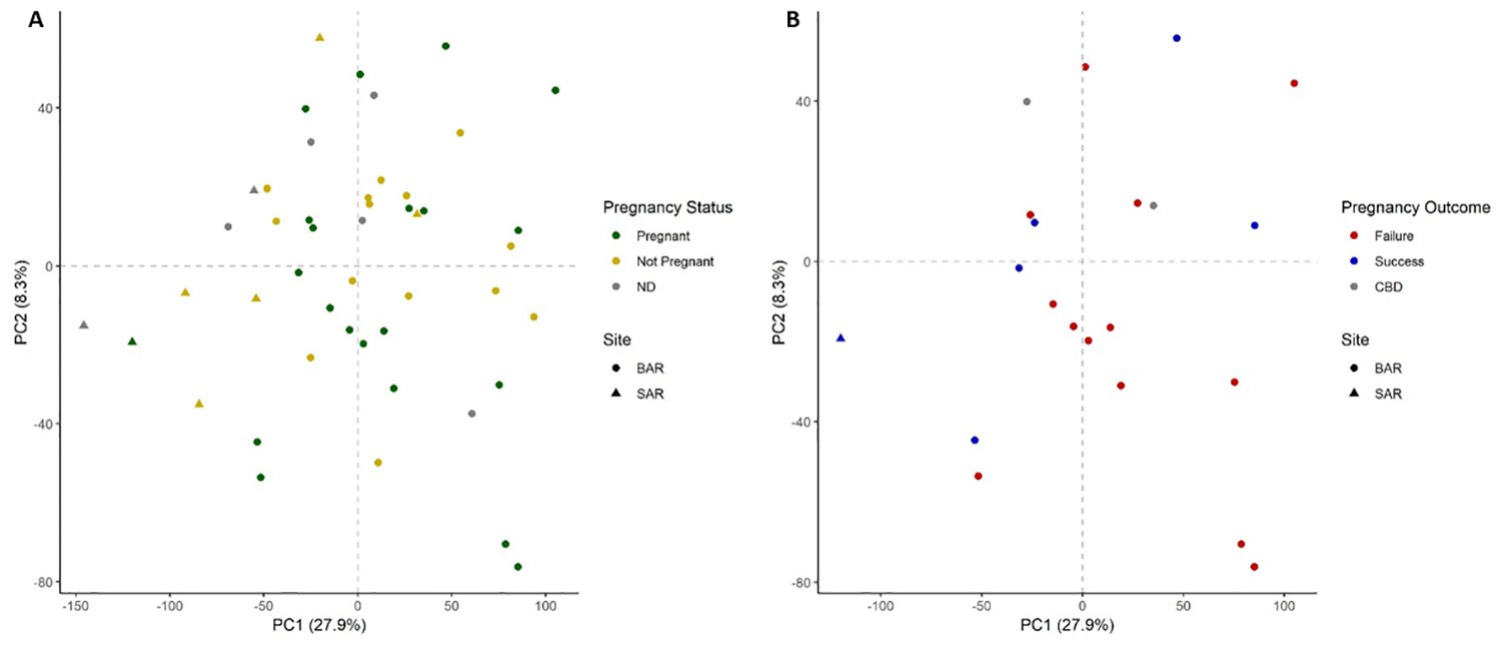
Principal component analysis of 11,500 expressed genes identified in the blood transcriptome of female dolphins from Sarasota Bay, FL (SAR) and Barataria Bay, LA (BAR). Principal component 1 accounted for 27.9% of variability and PC2 for an additional 8.3%. Transcript profiles did not cluster by pregnancy status (A) or outcome (B). Only pregnant dolphins are plotted on panel B. ND: data not available, CBD: cannot be determined.

FPKM values were filtered and 11,550 “expressed” genes were used for analysis. Transcript profiles did not clearly cluster by pregnancy status (Fig 6A) or pregnancy outcome in PCA (Fig 6B). Correspondingly, minimal differential expression was observed with only 37 genes (0.32%) different between pregnant (n = 21) and non-pregnant females (n = 20) (S8 Table) and no differences in gene expression between dams with successful (n = 6) and failed (n = 13) pregnancies. The majority of genes differentially expressed with pregnancy status (70%) were expressed more highly in non-pregnant females and are primarily involved in immune responses, particularly antiviral roles. While the limited number of changing genes here does not identify specific modulation of immune response, the previously discussed shift towards a Th17 response observed in BAR dolphins, relative to SAR, has been associated with adverse pregnancy outcomes in humans [30].

Ten annotated genes were more highly expressed in pregnant females, albeit with relatively minor fold-changes (1.4–2.2 fold). Many of these genes upregulated in pregnant females are known to have pregnancy-associated roles in other mammals including CDA, which, in humans, has been included in biomarker panels to assess endometrial receptivity in assisted reproduction [88]. Other genes of note are CCL2, an immune related gene that exhibited elevated transcript levels late in the gestation of rats [89] and CD8B, which demonstrated increased expression early in bovine pregnancy [90]. As most of the pregnancies in this study were first trimester, we cannot determine if expression in dolphins is stage-specific. However, with further research, expression of CCL2, CD8B, or expression ratios of the two genes may prove useful in approximating pregnancy stage, and thus broadly estimate due dates, in the absence of ultrasound data.

Also of note among the genes upregulated in pregnant females are GAS1 and LGALS1, both of which are involved in growth and cell proliferation processes and have known roles in pregnancy. Adequate expression of GAS1 is required for ovulation and luteinization in mice [91], possibly underlying the increased expression observed in our study. Substantial research has investigated the role of LGALS1 in fertilization, implantation, placentation, and stages of pregnancy. LGALS1 is increased by estrogen and progesterone and LGALS1-null mice show increased fetal loss [reviewed in 92, 93]. In light of substantial reproductive failures in BAR, LGALS1 gene expression may warrant additional research as circulating protein levels have been proposed as a biomarker of pre-eclampsia, miscarriage, and recurrent fetal loss [reviewed in 92].

One quarter of annotated genes differentially expressed between pregnancy status are mitochondrial-associated (NDUFS5, NDUFC2, GRAMD4). Of note is NDUFC2, an accessory subunit of the mitochondrial respiratory chain Complex I that, in human studies, is upregulated in response to triiodothyronine (T3) [94]. The relationship is likely similar in dolphins as WGCNA of female blood transcriptomes placed NDUFC2 in a gene module positively correlated with T3 serum concentrations (r = 0.47, p = 0.03, data not shown). In this study, NDUFC2 was expressed 1.4-fold higher in pregnant dams, but not correlated with pregnancy outcome. Single nucleotide polymorphisms (SNPs) in NDUFC2 are associated with abruption placentae caused by perturbations in OXPHO [95] and mitochondrial dysfunction is strongly associated with pregnancy failure and other reproductive issues [reviewed in 96, 97]. More broadly, research has indicated that reduced fertility and adverse pregnancy outcome associated with thyroid disorders may be due to mitochondrial dysfunction caused by transcriptional alterations in genes involved with OXPHO [94]. Thus the potential role of thyroid disorders and mitochondrial dysfunction in pregnancy failure is of great interest for the BAR dolphin population and changes in OXPHO and related gene expression in pregnancy failure may be an area of further research in the development of markers for predicting pregnancy outcome.

## Conclusions

Transcriptome profiling of blood from BAR and SAR dolphins identified a broad range of functional pathways that exhibited correlation to many standard health measures. Overall, gene expression was higher in BAR; however, transcripts associated with the ribosome and translation were more highly expressed in SAR. Elevated expression of transcripts associated with Th2-based immune responses were seen in BAR, driven by gene expression of dolphins likely exposed to significant oiling following the DWH disaster. Alterations in cytoskeletal gene expression were also observed between pre- and post-spill birth cohorts. Perturbations in OXPHO were abundant in BAR dolphins and may contribute to underlying molecular mechanisms associated with many observed adverse health and reproductive effects. The broad transcriptome profiling described herein and analyzed in conjunction with the array of health data obtained from capture-release health assessments is a fundamental step forward in developing molecular markers of health and exposure that can be applied to enhance assessment and characterization of stress-related disease in dolphins. With further research, biomarkers may be developed that can provide critical health information to wildlife veterinarians, researchers, managers and other stakeholders, even in the absence of full veterinary assessments.

## Supporting information

S1 Table Samples analyzed by RNA-seq and summarized health data. (XLSX)

S2 Table Genes in each co-expressed module from WGCNA of all BAR and SAR blood transcriptomes. The grey module, containing all genes not assigned to a co-expressed module is not included.
(XLSX)

S3 Table Genes exhibiting significant (p ≤ 0.05) differential expression between SAR and BAR. (XLSX)

S4 Table Genes in each co-expressed module from WGCNA of blood transcriptomes from BAR dolphins. The grey module, containing all genes not assigned to a co-expressed module is not included.
(XLSX)

S5 Table Genes exhibiting significant (p ≤ 0.05) differential expression between years in BAR, analyzed as a time course. (XLSX)

S6 Table Genes exhibiting significant (p ≤ 0.05) differential expression between years in BAR, all pairwise comparision analyzed. (XLSX)

S7 Table Genes exhibiting significant (p ≤ 0.05) differential expression between BAR dolphins known to be alive at the time of DWH oil spill and those born after. (XLSX)

S8 Table Genes exhibiting significant (p ≤ 0.05) differential expression between pregnant and non-pregnant females. (XLSX)
